# First field and laboratory evaluation of LAMP assay for malaria diagnosis in Cubal, Angola

**DOI:** 10.1186/s13071-023-05942-7

**Published:** 2023-10-03

**Authors:** Begoña Febrer-Sendra, Beatriz Crego-Vicente, Arlette Nindia, Joan Martínez-Campreciós, Sandra Aixut, Alejandro Mediavilla, Aroa Silgado, Inés Oliveira-Souto, Fernando Salvador, Israel Molina, Antonio Muro, Elena Sulleiro, Pedro Fernández-Soto

**Affiliations:** 1 Infectious and Tropical Diseases Research Group (e-INTRO), Biomedical Research Institute of Salamanca-Research Centre for Tropical Diseases at the University of Salamanca (IBSAL-CIETUS), Salamanca, Spain; 2 Hospital Nossa Senhora da Paz, Cubal, Angola; 3 Centro de Investigación Biomédica en Red de Enfermedades Infecciosas (CIBERINFEC), Instituto de Salud Carlos III, Madrid, Spain; 4 Microbiology Department Vall d’Hebron University Hospital, PROSICS Barcelona, Barcelona, Spain; 5 International Health Unit Vall d’Hebron-Drassanes, Infectious Diseases Department, Vall d’Hebron University Hospital, PROSICS Barcelona, Barcelona, Spain

**Keywords:** *Plasmodium* spp., Malaria, Loop-mediated isothermal amplification (LAMP), Field conditions, Point of care (POC), Angola

## Abstract

**Background:**

Malaria is a globally distributed infectious disease. According to the World Health Organization, Angola is one of the six countries that account for over half the global malaria burden in terms of both malaria cases and deaths. Diagnosis of malaria still depends on microscopic examination of thin and thick blood smears and rapid diagnostic tests (RDTs), which often lack analytical and clinical sensitivity. Molecular methods could overcome these disadvantages. The aim of this study was to evaluate, for the first time to our knowledge, the performance of a loop-mediated isothermal amplification (LAMP) for the diagnosis of malaria in an endemic area in Cubal, Angola, and to assess the reproducibility at a reference laboratory.

**Methods:**

A total of 200 blood samples from patients attended at Hospital Nossa Senhora da Paz, Cubal, Angola, were analysed for *Plasmodium* spp. detection by microscopy, RDTs, and LAMP. LAMP assay was easily performed in a portable heating block, and the results were visualized by a simple colour change. Subsequently, the samples were sent to a reference laboratory in Spain to be reanalysed by the same colorimetric LAMP assay and also in real-time LAMP format.

**Results:**

In field tests, a total of 67/200 (33.5%) blood samples were microscopy-positive for *Plasmodium* spp., 98/200 RDT positive, and 112/200 (56%) LAMP positive. Using microscopy as reference standard, field LAMP detected more microscopy-positive samples than RDTs (66/67; 98% vs. 62/67; 92.5%). When samples were reanalysed at a reference laboratory in Spain using both colorimetric and real-time assays, the overall reproducibility achieved 84.5%.

**Conclusions:**

This is the first study to our knowledge in which LAMP has been clinically evaluated on blood samples in a resource-poor malaria-endemic area. The colorimetric LAMP proved to be more sensitive than microscopy and RDTs for malaria diagnosis in field conditions. Furthermore, LAMP showed an acceptable level of reproducibility in a reference laboratory. The possibility to use LAMP in a real-time format in a portable device reinforces the reliability of the assay for molecular diagnosis of malaria in resource-poor laboratories in endemic areas.

**Graphical Abstract:**

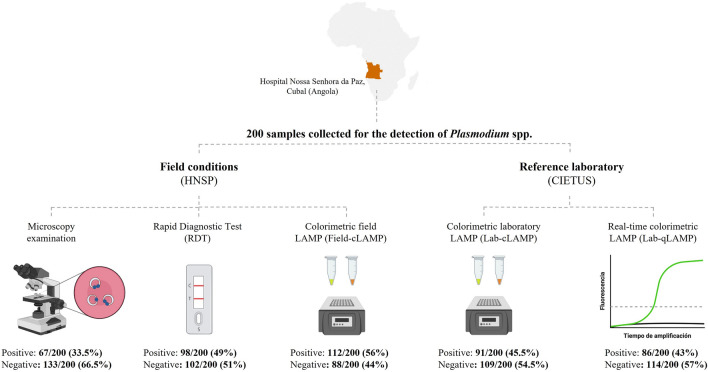

**Supplementary Information:**

The online version contains supplementary material available at 10.1186/s13071-023-05942-7.

## Background

Malaria is a vector-borne disease caused by *Plasmodium* protozoa and transmitted through the bite of infected female mosquitoes from the genus *Anopheles* [1]. Of more than 120 *Plasmodium* species infecting mammals, birds, and reptiles, only six are known to infect humans: *Plasmodium falciparum*, *P. vivax*, *P. malariae*, *P. ovale curtisi, P. ovale wallikeri* and *P. knowlesi* [2]. According to the World Health Organization (WHO), malaria is endemic in 84 countries, and the estimated number of malaria cases increased from 245 million cases in 2020 to 247 million in 2021. The major increase was observed in the WHO African Region [3]. *Plasmodium falciparum* and *P. vivax* are the predominant species worldwide, and the great majority of malaria cases caused by *P. falciparum* occur in sub-Saharan Africa (approximately 190 million cases) [2].

Malaria symptoms can be separated into two disease presentations: uncomplicated and severe malaria. Uncomplicated malaria symptoms are non-specific and include fever, chills, body aches, headache, cough, and diarrhoea, making clinical diagnosis unreliable. However, severe malaria can produce acute lung injury, respiratory distress syndrome, acute kidney injury, and acidosis [2]. Therefore, an early and accurate diagnosis of malaria is required to establish a therapy and avoid the risk of developing severe malaria. Currently, the gold standard method for malaria diagnosis remains microscopic examination of thin and thick blood smears [2, 4]. Unfortunately, microscopy examination has many limitations. It is time-consuming and requires expert training in parasite morphology, and false-negative results can occur when parasitaemia is low [4]. Other diagnostic methods widely used in endemic areas where good-quality microscopy cannot be maintained are rapid diagnostic tests (RDTs) based on the immunochromatographic detection of parasite-specific antigens circulating in the bloodstream. *Plasmodium falciparum* histidine-rich protein-2 (*Pf*HRP2), *P. falciparum* parasite lactate dehydrogenase (*Pf*-pLDH), *P. vivax* specific pLDH (*Pv-*pLDH), common human *Plasmodium* LDH (pan-pLDH), and aldolase are the most commonly used targets in malaria RDTs [4]. The RDTs are rapid, easy to use, and simple to interpret; nevertheless, they do not allow quantification of parasite load and sensitivity for detection of *P. vivax* is low and for *P. ovale* and *P. malariae* is poor [4–6]. Poor performance in the detection of *P. ovale* and *P. malariae* may be due to low affinity of some monoclonal antibodies to these species [7]. Also, false-negative results can occur because of heterogeneity in or deletion of the HRP2 gene [8]. For all this, RDT should be supported by other diagnostic methods to confirm *Plasmodium* infection.

At present, PCR-based methods such as nested and real-time PCR are widely used at reference laboratories providing high accuracy and sensitivity and can diagnose mixed infections [9]. However, these methods are costly, requiring sophisticated equipment and professional technicians, making them difficult to use in many regions where malaria is endemic. One of the most recent approaches in molecular diagnostics is the loop-mediated isothermal amplification (LAMP), a simple, rapid, specific, and cost-effective technique compared to PCR assays [10, 11]. LAMP works under isothermal conditions (demanding minimal infrastructure) employing the *Bacillus stearothermophilus* (*Bst*) DNA polymerase with strand displacement activity and using a minimum of four, and up to six, specially designed primers [10]. LAMP results can be visualized by colorimetric change or turbidity [12, 13] making it a suitable tool for use in low-resource settings where malaria is endemic. The first specific LAMP assay for the detection of *P. falciparum* was described in 2006 [14]. Since then, several in-house LAMP assays and commercial LAMP kits for malaria diagnosis have been developed [15]. The Eiken Loopamp™ Malaria Pan detection kit (Eiken Chemical Company, Tokyo, Japan) and the Illumigene Malaria Plus test (Meridian Bioscience Inc., Cincinnati, OH, USA) have been used successfully in numerous studies [16–20] and are the most widely used commercial LAMP kits for detecting malaria [21].

In Angola, a record of 3.7 million new cases and 5573 deaths were registered during the first 5 months of 2021 [22]. *Plasmodium falciparum* is responsible for more than 90% of malaria infections and remains among the top causes of mortality together with HIV/AIDS and tuberculosis, children under 5 years of age and pregnant women being the most vulnerable populations [1, 23]. To date, despite high prevalence of malaria in Angola, no studies have been conducted to demonstrate the efficacy of LAMP assays in the detection of *Plasmodium* spp. In this study, we evaluate a simple colorimetric LAMP assay previously described by Chen et al. [21] under field conditions in a low-income malaria-endemic area in Angola. The efficacy of this LAMP assay is compared with microscopy examination of thick blood smears as gold standard reference and also with the RDTs. Furthermore, the reproducibility of the colorimetric LAMP was evaluated in a reference laboratory.

## Methods

### Study area, population, and blood samples collection

**Fig. 1:**
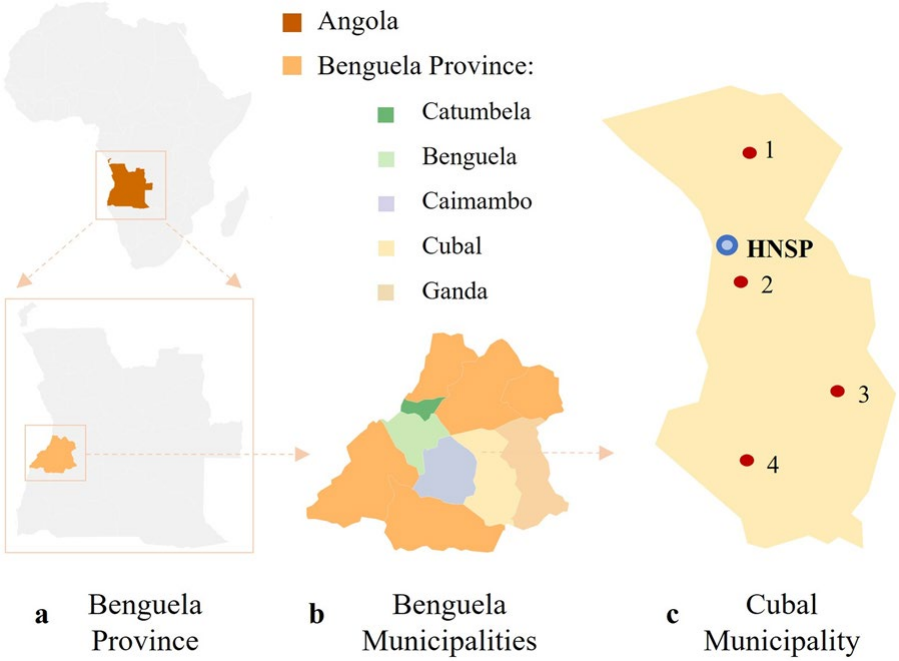
Map of Angola indicating Benguela Province, Benguela municipalities, and Cubal municipality. **a** Benguela Province. **b** Benguela municipalities (Catumbela, Benguela, Caimambo, Cubal, and Ganda) which participated in this study. **c** Cubal municipality: red dots indicate the urban communes (1 = Tumbulo; 2 = Cubal Sede; 3 = Yambala: 4 = Capupa) which participated in this study; blue dot indicates the position of Hospital Nossa Senhora da Paz (HNSP). Map was made using Datawrapper free software available online (https://www.datawrapper.de/) and Microsoft PowerPoint program

The study was conducted between May and July 2022 in the district of Cubal, Benguela Province, Angola, Africa, with an estimated population of 287,931 inhabitants [24]. Patients included in the study were attended at Hospital Nossa Senhora da Paz (HNSP) (Cubal Sede) and came from Benguela municipalities (Catumbela, Benguela, Caimambo, Cubal, and Ganda) and Cubal urban communes (Yambala, Capupa, Cubal Sede, and Tumbulo). A map of Angola indicating Benguela municipalities and Cubal urban communes is shown in Fig. 1.

The study population included a total of 200 patients (46% male; 56% female) attended at HNSP, and selection criterion was fever (≥ 37, 5 ºC). If for any reason, in any particular case, body temperature could not be taken, a febrile sensation or a clinical manifestation compatible with malaria diagnosed by an expert clinician was considered instead. Sociodemographic (age, gender, commune of residence) and clinical data (including fever, chills, body aches, and headache) were requested in a questionnaire with prior consent (Additional file 1: Table S1).

Blood samples were collected in the morning at the laboratory of HNSP. For each patient a fresh capillary blood specimen (for thin and thick blood films and to perform RDTs) and 3 ml of venous blood using an EDTA anti-coagulated tube (for further molecular analysis by LAMP) were obtained.

### Microscopy examination.

Microscopy examination was performed using fresh capillary blood. Thin and thick blood films were stained with 10% Giemsa for 15 min in staining jars. All slides were read by trained microscopist under 100× magnification with immersion oil. Parasite load was calculated in thick films according to the numbers of parasites per 100 leukocytes, assuming constant concentration of 8000 leukocytes/µl blood, including parasitic forms compatible with *P. falciparum* and also with *Plasmodium* no-*falciparum*. According to Alger et al. [25], a slide was classified as negative if no parasitic form of *Plasmodium* spp. was found after counting 500 leukocytes. Parasite load from microscopy-positive films was divided into three groups following methodology described by Fox et al. [26] as follows: low density (< 800 parasites/µl), moderate density (800–4000 parasites/µl), and high density (> 4000 parasites/µl).

### Malaria rapid diagnostic tests

The malaria rapid diagnostic test (RDT) used in this study was STANDARD™ Q Malaria P.f/Pan Ag Test (SD Biosensors, Republic of Korea) according to the manufacturers’ instructions using fresh capillary blood of patients. This RDT is a membrane-based immunochromatography for the qualitative detection of *P. falciparum*-specific Histidine Rich Protein 2 (HRP-2) and *Plasmodium* species (*P. falciparum, P. vivax, P. ovale, and P. malariae*) specific *Plasmodium* lactate dehydrogenase (pLDH). Results were obtained after 15 min.

### DNA extraction from blood samples

DNA extraction was performed on the day of sample collection. Aliquots of 200 µl venous whole blood were used for DNA extraction using NZY Tissue gDNA Isolation kit (NZYTECH, Lisbon, Portugal) following the manufacturer's instructions. DNA was eluted with 100 µl elution buffer.

Two aliquots of 50 µl each from extracted DNA samples were prepared and stored at − 20 °C in HNSP laboratory. One aliquot was used for LAMP assays, and the other was later shipped to our laboratory at the Center for Research in Tropical Diseases of the University of Salamanca (CIETUS, Salamanca, Spain) once the study was completed. DNA was extracted and stored as blood samples were collected so that the first DNA samples obtained were stored for longer (approximately 3 months) than those obtained at the end of the study (approximately 3 weeks) before being shipped to the laboratory in Spain.

### LAMP assay for *Plasmodium* spp. diagnosis

Colorimetric field-LAMP assay (field-cLAMP) was carried out at HNSP using the set of primers previously described by Chen et al. [21], targeting a portion of mitochondrial DNA (mtDNA) among *Plasmodium* spp. Briefly, field-cLAMP was carried out in a volume of 15 µl containing 1.6 µM of each FIP/BIP primer, 0.2 µM of each F3/B3, 0.4 µM of each LF/LB primer, and 0.6 µl of *Bst* 2.0 Warm Start DNA polymerase (New England Biolabs Ltd., Hitchin, UK) with 2 µl of template purified DNA. Reactions were incubated at 65 °C for 55 min in a portable heating block (AccuBlock ™ mini-compact, Labnet, Madrid, Spain) followed by heating at 80 °C for 5–10 min to stop the reaction. LAMP results were visually detected by colour change (green: positive; orange: negative) by adding 2 µl of 1:10 diluted 10,000× concentration SYBR Green I (Invitrogen, Waltham, MA, USA) in each tube.

DNA from a malaria-positive patient sample confirmed by qPCR (hereafter, C +), provided by the Department of Microbiology, Vall d´Hebron University Hospital, Barcelona, Spain, was used as positive control. Ultrapure water was used as negative control in field-cLAMP assays. Amplification assays were performed in batches of 10 samples each and each batch included one positive and one negative control.

At reference laboratory, the colorimetric LAMP assay (lab-cLAMP) and real-time LAMP assay (lab-qLAMP) were performed using the same primers set and reaction conditions as mentioned for field-cLAMP assay.

For lab-qLAMP assay, 0.24 µl EvaGreen 20× (Biotium, San Francisco, CA, USA) was added to monitor the fluorescence in real time. Reactions were performed in an Eco48 real-time PCR system (PCRmax, Beacon Road, Stone, Staffordshire, UK) programmed at 65 °C for 55 min followed by 10 min at 80 °C to stop the reaction. Amplification assays were performed in batches of 20 samples each for easy handling and to avoid cross-contamination. Same positive and negative controls were used as mentioned above.

All LAMP tests carried out in the field and in the reference laboratory were performed and tested by the same experienced researcher.

### Statistical analyses

The clinical sensitivity, specificity, positive predictive value (PPV), and negative predictive value (NPV) of RDTs, field-cLAMP, lab-cLAMP, and lab-qLAMP assays were determined by considering microscopy as the gold standard method for diagnosis. A Cohen’s kappa coefficient was performed to evaluate the concordande between field-cLAMP/lab-cLAMP and field-cLAMP/lab-qLAMP. The confidence intervals (CI) were established at 95%. All values were calculated using the free WinEpi 2.0 software [27].

## Results

### Microscopy, RDTs and LAMP under field conditions

*Plasmodium* spp. infections were detected by microscopy in 67/200 (33.5%) thick films. Parasite load is shown in Table 1. The highest percentage of microscopy-positive films was included in the group with the highest parasite load (32/67; 47.7%, > 4,000 parasites/µl).

**Table 1:** Parasite load from a total of 67 microscopy-positive films divided into three groups according to methodology described by Fox et al. [26] and percentage from each group

Parasite load	Positive samples (N = 67)	Percentage
Low density (< 800 parasites/µl)	20	29.8
Moderate density (800–4000 parasites/µl)	15	22.4
High density (> 4000 parasites/µl)	32	47.7

HRP-2/pLDH based RDTs detected 98/200 (49%) positive samples. A total of 62/67 (92.5%) microscopy-positive samples were also positive by RDTs. However, RDT did not detect 5 microscopy-positive with low and moderate density (400, 160, 160, 5866, 962 parasites/µl). Considering microscopy-negative samples, 97/133 (72.9%) were also negative by RDT.

By field-cLAMP assay we obtained positive results in 112/200 (56%). Of the 67 microscopy-positive thick films samples, 66 (98.5%) were LAMP positive. Only one sample with low parasitic load (650 parasites/µl) was missed. Of the 133 microscopy-negative thick films, 87 (65.4%) were also negative by field-cLAMP.

**Fig. 2:**
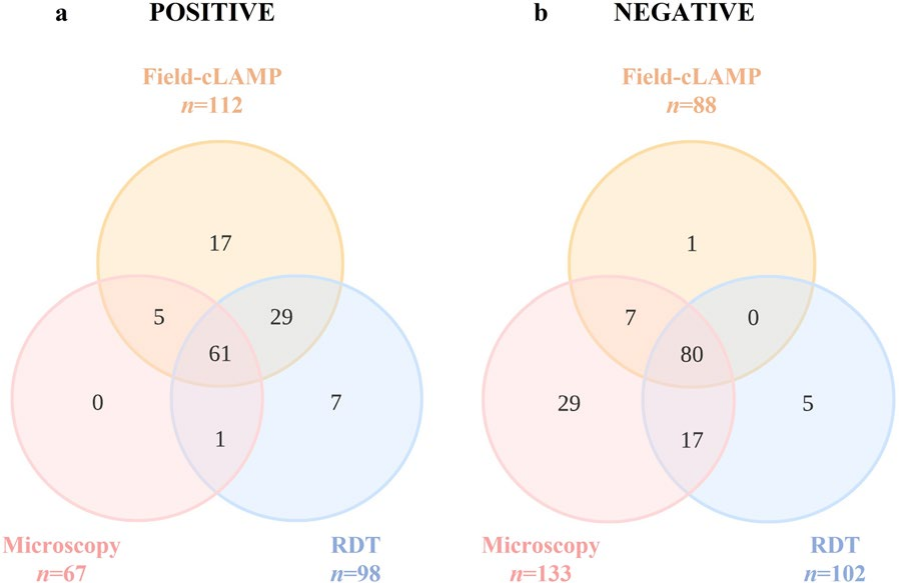
Venn diagrams for comparison of microscopy, RDT, and field-cLAMP in field trials. **a** Distribution of the samples with *Plasmodium* spp.-positive results for at least one test. **b** Samples with *Plasmodium* spp.-negative results for at least one test. RDT, rapid diagnostic test; Field-cLAMP, colorimetric field LAMP assay

The results of RDTs and field-cLAMP assays in field trials were compared with microscopy as the reference standard and overlaps of all are shown using Venn diagrams in Fig. 2. Notably, up to 61 of the 67 (91.1%) samples with microscopy-positive results were positive by the three methods (Fig. 2a). A total of 80/133 samples (70.8%) with a microscopy-negative result were also negative for all detection methods performed (Fig. 2b).

### LAMP and qLAMP assays at a reference laboratory

Lab-cLAMP assay detected *Plasmodium* spp. in 91/200 (45.5%) samples. Overall, 60/67 (89.6%) microscopy-positive samples were also positive by lab-cLAMP. Surprisingly, lab-cLAMP assay did not detect seven microscopy-positive samples with high parasite load (400, 160, 36,900, 1103, 5866, 650, and 674 parasites/µl). Considering microscopy-negative samples, 102/133 (76.7%) were negative.

A total of 86/200 (43%) positive results were obtained using lab-qLAMP assay. Lab-qLAMP detected 58/67 (86.6%) microscopy-positive results. However, lab-qLAMP was not able to amplify nine microscopy-positive samples with a high parasite load (400, 160, 160, 36,900, 1103, 500, 5866, 650, and 674 parasites/µl). Regarding to negative samples, 105/133 (78.9%) of microscopy-negative samples were also negative by lab-qLAMP assay.

### Field tests versus reference laboratory assays

The results obtained with RDTs, field-cLAMP, lab-cLAMP, and lab-qLAMP assays to detect *Plasmodium* spp. compared with microscopy as the reference standard are shown in Table 2. Field-cLAMP assay showed the highest sensitivity (98.5%) whereas lab-qLAMP showed the best specificity (78.9%).

**Table 2:** Results obtained by diagnostic methods used in this study in comparison with microscopy as reference standard

	Total	Microscopy	
		Positive (*N* = 67)	Negative (*N* = 133)
RDT +	98	62 (92.5%)	36 (27.1%)
RDT−	102	5 (7.5%)	97 (72.9%)
Field-cLAMP +	112	66 (98.5%)	46 (34.6%)
Field-cLAMP−	88	1 (1.5%)	87 (65.4%)
Lab-cLAMP +	91	60 (89.6%)	31 (23.3%)
Lab-cLAMP−	109	7 (10.4%)	102 (76.7%)
Lab-qLAMP +	86	58 (86.6%)	28 (21.1%)
Lab-qLAMP−	114	9 (13.4%)	105 (78.9%)

RDT + and RDT-, positive and negative results by Rapid Diagnostic Tests; Field-cLAMP + and Field-cLAMP-, positive and negative results detected by colorimetric field LAMP assay; Lab-cLAMP + and Lab-cLAMP-, positive and negative results obtained by colorimetric laboratory LAMP assay; Lab-qLAMP + and Lab-qLAMP-, positive and negative results amplified by laboratory real-time LAMP assay

Nevertheless, when diagnostic parameters were calculated using microscopy as reference standard, we obtained the lowest PPV (58.9%) using the field-cLAMP assay (Table 3).

**Table 3:** Estimation of sensitivity, specificity, and positive and negative predictive values of diagnostic methods used in this study to detect *Plasmodium* spp. using microscopy as reference

	RDT	Field-cLAMP	Lab-cLAMP	Lab-qLAMP
Sensitivity (95% CI)	92.5% (86.2%, 98.8%)	98.5% (95.6%, 101.4%)	89.6% (82.2%, 96.9%)	86.6% (78.4%, 94.7%)
Specificity (95% CI)	72.9% (65.4%, 80.5%)	65.4% (57.3%, 73.5%)	76.7% (69.5%, 83.9%)	78.9% (72.0%, 85.9%)
PPV (95% CI)	63.3% (53.7%, 72.8%)	58.9% (49.8%, 68.0%)	65.9% (56.2%, 75.7%)	67.4% (57.5%, 77.3%)
NPV (95% CI)	95.1% (90.9%, 99.3%)	98.9% (96.6%, 101.1%)	93.6% (89.0%, 98.2%)	92.1% (87.2%, 97.1%)

RDT, Rapid Diagnostic Tests; Field-cLAMP, colorimetric field LAMP assay; Lab-cLAMP, colorimetric laboratory LAMP assay; Lab-qLAMP, laboratory real-time LAMP assay; PPV, positive predictive value; NPV, negative predictive value; CI, confidence intervals

**Fig. 3:**
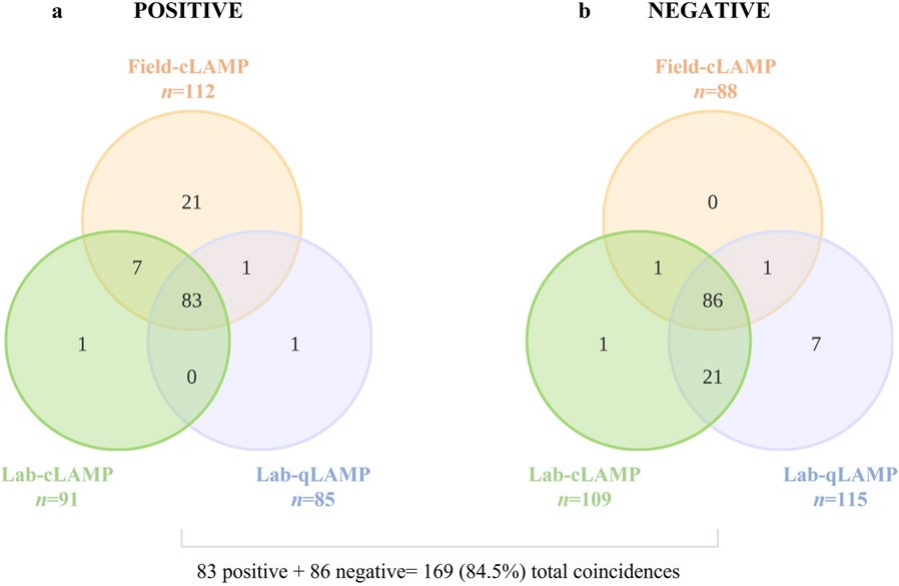
Venn diagrams for three-way comparison of field-cLAMP, lab-cLAMP, and lab-qLAMP assays. **a** Distribution of the samples with *Plasmodium* spp.-positive results for at least one LAMP test. **b** Distribution of the samples with *Plasmodium* spp.-negative results for at least one LAMP test. Field-cLAMP, colorimetric field LAMP assay; Lab-cLAMP, colorimetric laboratory LAMP assay; Lab-qLAMP, real-time laboratory LAMP assay

The overlaps of the three resulting LAMP assays are shown using Venn diagrams (Fig. 3). A total of 83 samples were positive (Fig. 3a) and 86 samples were negative (Fig. 3b) by the three LAMP assays, resulting in an 84.5% match rate. A 77.2% (kappa 0.77) and 71.5% (kappa 0.71) concordance between field-cLAMP/lab-cLAMP and field-cLAMP/lab-qLAMP, respectively, was obtained.

## Discussion

At present, the thick and thin blood smear examination by light microscopy remains the reference method for malaria diagnosis throughout the world [28]. This method has some advantages such as low cost, differentiation of malaria species, and quantification of parasite load. However, it is a labour-intensive method whose quality strongly relies on experienced personal [4]. Therefore, ther has been interest in RDTs in combination with microscopy examination in many malaria-endemic areas with high prevalence [4, 28]. Nevertheless, microscopy and RDT methods are not sensitive enough to detect low density malarial infections [29]. LAMP technology could be a good molecular tool to solve these drawbacks due to the high sensitivity and not needing special equipment. In this study, with the aim of implementing molecular diagnosis of malaria under field conditions in an endemic area in Cubal, Angola, we evaluated a previously developed LAMP assay [21] and compared it with microscopy as a reference method and with commercial RDTs. In addition, results were subsequently compared in a reference laboratory to assess the reproducibility of the assay.

According to the World Malaria Report 2022, Angola is among the high-burden countries, accounting for 3.4% of all malaria cases worldwide in 2021 [3] and poses the biggest health threat to pregnant woman and children under 5 years of age [1]. The main health facility in Cubal is the HNSP, a referent hospital for infectious diseases in the area. However, few studies have focused on determining the prevalence of malaria in symptomatic patients in this rural area. A retrospective and observational study carried out at the HNSP between January 2009 and December 2013 showed a prevalence of 14.2% for *P. falciparum* using thick blood films as unique diagnostic method [23]. However, more recently, another retrospective study conducted between January 2014 and December 2016 showed an increase in the incidence of malaria in Cubal using both microscopy (27.2%) and RDTs (50.6%) [30]. Our study was conducted during May and July 2022 showing similar results for microscopy (33.5%) and RDTs (49%). Nevertheless, when we tested the blood samples by field-cLAMP assay, the overall of positive results increased significantly by up to 56%. Similar results were previously obtained using LAMP in high transmission malaria-endemic areas such as Uganda, Gambia, and Peruvian Amazon [16, 31, 32].

However, the use of antigen-detecting RDTs is a priority and essential for malaria diagnosis in areas where good-quality microscopy cannot be maintained [33]. Thus, the number of malaria RDTs and the scale of their use have increased (3.5 billion RDTs for malaria were sold in 2010–2021) with almost 82% of these sales being in sub-Saharan African countries [3]. However, false-negative results may occur in parasites that cannot express HRP2 as has been recently demonstrated in several studies carried out in African countries such as Ethiopia, Nigeria, Sudan, Madagascar, and Tanzania [34–38]. Additionally, the low sensitivity of the RDTs must also be considered [5]. Design limitations of RDTs include poor sensitivity at low parasite densities, susceptibility to the prozone effect (PfHRP2-detecting RDTs), false-negative results due to PfHRP2 deficiency in the case of pfhrp2 gene deletions (PfHRP2-detecting RDTs), cross-reactions between *Plasmodium* antigens and detection antibodies, false-positive results by other infections, and susceptibility to heat and humidity [5]. This could explain our RDT-negative results in five microscopy-positive samples, including a sample with moderate parasite load (5866 parasites/µl).

However, a total of 29 samples were positive only by both RDTs and field-cLAMP assays, with better sensitivity results using LAMP (98.5%) than RDTs (92.5%). Our results are in line with those obtained in a mass screening of asymptomatic malaria study carried out in Zanzibar [39]. The simplicity and high sensitivity of these techniques make them good tools to use in rural areas of Africa with poor sanitary conditions. Nonetheless, due to limitations, RDTs should be supported by other diagnostic methods to diagnose *Plasmodium* infection [4] such us LAMP technology.

Additionally, interlaboratory comparisons are needed to determine the reproducibility of analytical methods to be standardised. In this sense, the samples analysed in field conditions were reanalysed by LAMP in a reference laboratory. We obtained lower positive results using lab-cLAMP (45.5%) and lab-qLAMP (43%). Attending to microscopy-positive results, only one sample was not detected by the three LAMP assays (field-cLAMP, lab-cLAMP, and lab-qLAMP). This could be associated with a sample handling error when extracting the DNA. Unexpectedly, at the reference lab, both lab-cLAMP and lab-qLAMP assays failed to amplify *Plasmodium* spp. in seven microscopy-positive samples, including one with high parasite load (36,900 parasites/µl). We believe that this result is not related to the sensitivity of the LAMP assays because other samples with lower parasite load were LAMP positive. As is known, the freeze-thaw process of samples can affect the yield and integrity of DNA [40]. Thus, the decreased efficacy of both LAMP assays in the lab compared to the field could be due to long-time storage of samples, inadequate cold chain maintenance for preservation under field conditions, and subsequent shipment without refrigeration. A possible error in sample handling could not be ruled out either. Also, two samples were not amplified by lab-qLAMP, probably because of EvaGreen fluorescent dye in the reaction master mixes for real-time monitoring. It has been reported that EvaGreen can sometimes result in partial inhibition of the LAMP reaction and reduce the rate and final levels of amplification [41]. This inhibitory effect has been shown in other studies carried out by our group using other LAMP assays [42, 43]. Nonetheless, a very reasonable agreement of 84.5% was obtained between field-based LAMP and reference laboratory-based LAMP assays.

## Conclusions

In this field study, LAMP technology was used for the first time to our knowledge for the detection of *Plasmodium* spp. in a malaria-endemic area in Angola, demonstrating higher sensitivity than microscopy and RDTs. The ease of use, simplicity, and feasibility demonstrated by LAMP assay in field conditions together with the acceptable level of reproducibility achieved in a reference laboratory and possibility to use a real-time format in a portable device support the use of LAMP assay as an effective test for molecular diagnosis of malaria in resource-poor laboratories in endemic areas.

### Supplementary Information

**Additional file 1: Table S1.** Sociodemographic and clinical data of the 200 patients included in this study.

## Data Availability

All data generated or analysed during this study are included in this published article.

## Abbreviations

WHO: World Health Organization
RDT: Rapid diagnostic test
HRP2: Histidine-rich protein-2
pLDH: Parasite lactate dehydrogenase
PCR: Polymerase chain reaction
LAMP: Loop-mediated isothermal amplification
HNSP: Hospital Nossa Senhora da Paz
CIETUS: Center for Research in Tropical Diseases of the University of Salamanca
PPV: Positive predictive value
NPV: Negative predictive value
