# Supplementary material for: First field and laboratory evaluation of LAMP assay for malaria diagnosis in Cubal, Angola
Source: Parasit Vectors. 2023 Oct 3;16:343. doi: 10.1186/s13071-023-05942-7 (PMC10548721; doi:10.1186/s13071-023-05942-7)
Supplement: Supplementary file 1 — Additional file 1: Table S1. Sociodemographic and clinical data of the 200 patients included in this study. [file 13071_2023_5942_MOESM1_ESM.pdf]

Table S1 Sociodemographic and clinical data of the 200 patients included in this study.

Nº Patient	Gender	Age (years)	Urban communes/ Neighborhoods	Clinical data[a]
1	F	<1	Ngola	
2	M	48	Cristo Rei	
3	F	21	Capupa	
4	M	2	Cubal	
5	M	11	Cubal	
6	M	3	Cubal	
7	F	10	Cubal	
8	F	24	Cubal	
9	F	45	Cubal	
10	F	40	Ngola	
11	F	30	NA	Headache
12	F	1	NA	Malnutrition
13	M	2	Jamba	
14	M	46	Kasseke	
15	M	44	Passai	
16	M	<1	Bairro 80	Diarrhea
17	F	39	Cristo Rei	
18	F	18	NA	Headache
19	M	58	NA	Headache
20	F	3	Benfica	
21	M	26	Passagem	Headache
22	F	39	Kalohala	Abdominal pain
23	F	2	Camunda	
24	M	1	Calomanga	
25	F	40	Calomanga	Abdominal pain
26	F	29	NA	Headache
27	M	<1	Marco	
28	M	1	Calomanga	Abdominal pain
29	F	14	Ciudade	Headache
30	M	8	Lulambo	Abdominal pain

31	M	18	Bairro 80	
32	M	<1	Kasseke	Abdominal pain
33	M	4	NA	
34	F	77	Cubal	Body-aches
35	M	4	NA	
36	F	15	Camunda	Abdominal pain
37	M	21	Marco	
38	M	6	Cristo Rei	
39	F	6	Passagem	
40	F	30	Catumbela	Headache
41	F	<1	Kasseke	
42	F	12	Cristo Rei	Headache
43	F	27	Ngola	Headache
44	M	13	Camunda	Abdominal pain
45	F	45	Benguela	Body-aches
46	F	33	NA	
47	M	2	Ngola	
48	F	10	Capupa	
49	F	3	Kalohala	
50	M	1	Kombondongolo	Malnutrition
51	F	17	NA	Headache, abdominal pain
52	M	42	Cubal	
53	M	2	NA	Headache, body-aches
54	M	1	Cubal	
55	M	<1	Cerâmica	
56	F	1	Calomanga	
57	M	<1	Marco	Abdominal pain
58	F	15	Sagrada Familia	Abdominal pain
59	F	6	Lulambo	Body-aches
60	M	2	NA	
61	F	1	NA	
62	M	60	Kalohala	Body-aches
63	M	52	NA	
64	M	12	NA	

65	F	1	Yambala	Abdominal pain
66	M	9	Passai	Abdominal pain
67	M	1	Kassica	Anemia
68	M	16	Benfica	Headache
69	F	1	Caimambo	Malnutrition
70	F	2	Capupa	
71	F	7	Sagrada Familia	
72	F	6	NA	
73	M	34	Ngola	Headache
74	M	10	Passagem	
75	F	5	Benfica	Headache, abdominal pain
76	M	6	NA	
77	M	10	NA	
78	F	12	Yambala	Malnutrition
79	F	18	Ngola	Headache
80	F	1	NA	Headache
81	M	2	Caimambo	Headache
82	M	2	Calomanga	Diarrhea, headache
83	F	2	Calomanga	Abdominal pain, diarrhea
84	M	7	NA	Diarrhea
85	M	1	Bairro 80	
86	M	8	Cristo Rei	Headache
87	F	1	Ganda	Abdominal pain
88	F	45	Sagrada Familia	Headache
89	M	1	Yambala	
90	F	2	Yambala	
91	F	10	Camunda	
92	F	2	Ngola	
93	F	10	Sagrada Familia	
94	F	50	NA	
95	M	5	Ngola	
96	F	11	Ngola	
97	M	26	Benfica	Headache, body-aches
98	F	4	Lomaum	Headache

99	M	2	Lomaum	
100	F	9	Ngola	
101	F	NA	NA	Headache, body-aches
102	F	44	Passagem	
103	F	56	Bairro 80	Headache
104	F	42	Calomanga	Headache, abdominal pain
105	F	1	Yambala	
106	M	<1	Cerâmica	Anemia
107	F	41	Assunsao	Headache
108	F	5	Yambala	
109	F	1	NA	Malnutrition
110	F	10	Kalohala	Headache, abdominal pain
111	M	14	Sagrada Familia	Headache, abdominal pain
112	F	<1	Cristo Rei	Diarrhea
113	F	19	NA	Headache, abdominal pain
114	F	<1	Passagem	
115	F	1	Jamba	
116	M	34	Calomanga	
117	M	28	Lomaum	
118	F	2	Ngola	Abdominal pain
119	F	50	NA	
120	F	22	NA	
121	M	32	Cristo Rey	Body-aches, headache
122	M	<1	Halu	
123	M	38	Cubal	
124	F	31	Capupa	
125	F	51	Camunda	
126	F	47	NA	Headache
127	F	6	Halu	
128	F	50	Camunda	Headache, body-aches
129	F	50	Kalanda	
130	M	18	Calaiala	Body-aches
131	M	19	Calaiala	Body-aches
132	F	3	Halu	

133	M	37	Kasseke	Body-aches
134	F	2	Marco	Body-aches
135	M	17	Camunda	
136	F	<1	NA	
137	M	3	NA	
138	F	<1	Kassiva	
139	F	1	Tumbulu	
140	M	8	Cristo Rey	Headache
141	F	<1	Cristo Rey	
142	M	1	Camunda	
143	F	40	Marco	Headache, body-aches
144	M	64	Cubal	Body-aches
145	F	16	NA	Body-aches
146	F	37	Calomanga	Headache
147	M	3	Caimambo	Headache
148	M	2	Bundiangolo	
149	F	2	Sagrada Familia	
150	F	8	Yambala	
151	F	2	NA	
152	M	2	Ngola	
153	F	7	Cristo Rei	
154	M	5	Cristo Rei	
155	M	36	Benfica	Headache
156	F	<1	Passagem	
157	M	<1	Ngola	Abdominal pain
158	F	55	NA	
159	F	45	Benguela	Headache, abdominal pain
160	F	60	Tumbulu	Body-aches
161	M	18	Calomanga	Headache
162	M	30	Benguela	Headache, body-aches
163	F	22	Yambala	Body-aches
164	M	4	Benfica	Headache
165	M	2	Camunda	
166	M	1	Passagem	

167	F	5	Cristo Rey	Abdominal pain
168	M	1	NA	
169	M	1	Yambala	
170	F	55	NA	Headache, body-aches
171	M	35	Barrio 80	Headache, body-aches
172	M	23	Caimambo	Headache, abdominal pain
173	M	28	Ciudade	Body-aches
174	M	10	Cristo Rey/Cubal	
175	F	<1	Assunsao	
176	F	<1	Caimambo	Diarrhea
177	M	21	Capupa	Headache
178	F	1	Bairro 80	
179	F	9	Bairro 80	Malnutrition
180	M	1	Ciudade	
181	F	<1	NA	
182	F	1	Ngola	Malnutrition
183	F	32	Camunda	Headache
184	M	33	Calomanga	
185	M	<1	NA	Abdominal pain
186	F	<1	NA	
187	F	1	Chimboa	
188	M	<1	NA	
189	F	23	Hambi	
190	M	4	Camunda	
191	M	3	Kasseke	Body-aches
192	M	9	Ngola	Headache
193	F	1	Kasseke	
194	M	1	Kasseke	Abdominal pain
195	M	1	Kasseke	
196	M	9	Bonda	
197	F	<1	Bairro 80	
198	M	2	NA	Abdominal pain
199	F	<1	NA	
200	F	1	Tumbulu	

[a]Clinical data: all patients had a fever of 37.5ºC or higher. If for any reason, in any particular case, body temperature could not be taken, a febrile sensation or a clinical manifestation compatible with malaria diagnosed by an expert clinician was considered instead.; F: female; M: male; NA: not available.
